# Deciphering morphological and biochemical modulations in mungbean by application of ethylene precursor and inhibitors under cadmium stress

**DOI:** 10.1038/s41598-025-96375-y

**Published:** 2025-05-01

**Authors:** Aneeqa Munawar, Muhammad Sohail Akram, Shafaqat Ali, Pallab K. Sarker, Mohamed A. El-Sheikh

**Affiliations:** 1 Department of Botany, Government College University Faisalabad, Faisalabad, 38000 Pakistan; 2 Department of Environmental Sciences, Government College University Faisalabad, Faisalabad, 38000 Pakistan; 3 Department of Biological Sciences and Technology, China Medical University, Taichung, 40402 Taiwan; 4 Environmental Studies Department, University of California Santa Cruz, Santa Curz, CA 95060 USA; 5 Botany and Microbiology Department, College of Science, King Saud University, Riyadh, Saudi Arabia

**Keywords:** 1-amino cyclopropane-1-carboxylic acid, Cadmium, Ethylene, Inhibitors, Mungbean, Environmental impact, Biochemistry

## Abstract

Cadmium (Cd), being one of the most toxic elements, exerts negative effects on plant growth and development. Ethylene (ET) has a prime significance in abiotic stress-induced cellular modulations and regulation of plant development. In the present study, seeds of a Cd-tolerant (NM-98) and -sensitive (NM-28) genotype were primed using 50 µM ACC (ET precursor 1-amino cyclopropane-1-carboxylic acid) and ET inhibitors (1 mM AVG/aminoethoxyvinylglycine, 0.5 mM PZA/pyrazinamide or 0.25 mM AgNO_3_/silver nitrate). Subsequently, these were allowed to grow in soil containing 0 or 50 µM CdCl_2_. After germination, ACC, AVG, PZA or AgNO_3_ was again applied, accordingly, as foliar spray. In NM-98, application of ACC resulted in reduced root fresh (38%) and dry (38%) weight, shoot fresh weight (39%), total phenolics (22%), catalase/CAT and peroxidase/POD activity (21% and 16%, respectively) under Cd stress. Contrarily, application of inhibitors (AVG, PZA and AgNO_3_), under Cd stress, improved plant biomass, photosynthetic pigments, total phenolics, total soluble proteins, activity of CAT and POD in NM-98 as compared to NM-28 genotype that exhibited opposite response. It was concluded that contrasting modulations observed in NM-98 and NM-28 towards exogenous ACC (or inhibitors) was linked with Cd-tolerance level of the two genotypes where exogenous ACC likely significantly raised endogenous ET level in NM-98 leading to disrupted antioxidants status and reduced growth. However, AVG, PZA and AgNO_3_ maintained ET levels and thus NM-98 exhibited less oxidative stress. Meanwhile, exogenous ACC assisted growth of NM-28 in Cd-stressed soil by modulating the antioxidative defence mechanism, possibly by maintaining endogenous ET concentrations. Further molecular-level research is recommended to investigate the underlying mechanisms associated with each component of ET biosynthesis.

**Supplementary Information:**

The online version contains supplementary material available at 10.1038/s41598-025-96375-y.

## Introduction

Heavy metals (HMs) are toxic environmental contaminants that threaten quality of soil and water, as well as plants and animal nutrition^1,2^. Cadmium (Cd) is mainly used as a reagent in laboratory experiments, in electroplating processes for corrosion protection, manufacturing batteries, and pharmaceuticals^2^. Cadmium-contaminated soils exerted negative impacts on various plant tissues/organs and slow down developmental processes^3,4^. Its uptake and translocation in plants occurs by various transporters including yellow stripe 1-like transporter (YSL), zinc-regulated and iron-regulated transporter-like protein (Zrt, Irt-like, ZIP), heavy metal ATPase (HMA), natural resistance-associated macrophage protein (NRAMP) and CAX transporter^5^. Evidence suggests that Cd poisoning causes oxidative stress, damages cellular membranes and electron transport, inhibits or activates enzymes, interferes with nucleic acids and photosynthesis, and stunts plant growth^4^. It may influence plasma membrane permeability and alter activity of nutrient transporters, preventing nutrients from being absorbed and changing their concentration and composition^3^. In order to protect themselves from various stress circumstances, plants employ a scavenging mechanism that includes both enzymatic and non-enzymatic antioxidants^4,5^.

Ethylene (ET) is a multifunctional plant hormone involved in growth, development, and stress responses. The biosynthesis of ET involves two committed enzymatic reactions. First, ACC synthase (ACS) catalyzes the conversion of S-adenosyl-L-methionine (SAM) into 1-aminocyclopropane-1-carboxylic acid (ACC) and 5′-methylthioadenosine (MTA)^6^. The ACC oxidase (ACO) subsequently converts ACC into ET, CO_2_, and cyanide^7,8^. 1-aminocyclopropane-1-carboxylic acid (ACC) was discovered in 1950^9^ and was initially considered just a precursor of ET. However, recent studies indicated that ACC itself may function as a signaling molecule that affects plant growth, stress reactions, and microbial interactions independently of ET^10^. In *Arabidopsis thaliana*, ACC has been demonstrated to regulate pollen tube attraction, vegetative development, guard cell differentiation and cell wall metabolism^8^. The reduction of root permeability, stomatal closure, aerenchyma development, adventitious roots formation, and early fruit loss caused by stress may be directly or indirectly related to the rise in endogenous ACC or its conjugates^8^.

Under stress conditions, ET production is often upregulated, leading to enhanced stress signaling and adaptive responses. However, excessive ET accumulation can exacerbate Cd-induced damage by promoting senescence, inhibiting root elongation, and increasing reactive oxygen species (ROS) levels^11^. To better understand and regulate ET role under Cd stress, the use of ET inhibitors has become a valuable approach in plant stress physiology research. Aminoethoxyvinylglycine (AVG) is a potent inhibitor of ACS, which catalyzes the conversion of S-adenosylmethionine (SAM) to 1-aminocyclopropane-1-carboxylic acid (ACC), the direct precursor of ET. AVG aid in understating the ET-mediated stress responses as it efficiently lowers ET production through ACS inhibition^12^. AVG further contributes to stress tolerance by influencing tryptophan aminotransferase activity which in turn affects other metabolic pathways including auxin production. AVG has been shown to inhibit ET accumulation in leaves, leading to enhanced leaf growth, improved nitrogen uptake, and better photosynthetic performance, ultimately boosting fruit production in cotton cultivars under waterlogged condition^12^. External application of AVG has also been found to lower plant respiration rates^13^. Its pre and postharvest use delays fruit ripening while improving the storage potential of several climacteric fruits^13^.

Pyrazinecarboxylic acid (POA) is produced from pyrazinamide (PZA) and binds to/inhibits ACO, the enzyme responsible for the last step of ET production^14^. Surprisingly, PZA can assist plant growth by reducing stress-induced cellular damage through regulating endogenous ET levels^14,15^. Under heavy metal stress, ET overproduction frequently leads to enhanced lipid peroxidation, early aging, and rapid leaf yellowing^17^. However, PZA treatment has been shown to lessen oxidative damage and delaying ET-mediated senescence under abiotic stress^16^. PZA-induced ET suppression can also enhance root growth, which is crucial for efficient water and nutrient uptake under abiotic toxicity^18^.

A common ET perception inhibitor, silver nitrate (AgNO₃), competes with ET for receptor binding, thereby blocking downstream ET-mediated stress responses^19^. Silver nitrate has been demonstrated to reverse ET-induced growth suppression and senescence under abiotic stress^18^. It helps to preserve chlorophyll content, enhance photosynthetic activity, and postpone stress-induced senescence by inhibiting ET sensing. Recent studies have demonstrated that AgNO_3_ can effectively counteract the effects of externally applied ET, preventing typical ET-induced responses such as wilting, senescence, and inhibited plant growth^20^. Similarly, another study reported that AgNO₃ suppressed ET biosynthesis, leading to the regeneration of multiple shoots from cotyledon and hypocotyl explants in cotton^21^. Additionally, AgNO_3_ treatment has been shown to reduce postharvest losses in guava fruits by delaying ripening, minimizing spoilage, and extending shelf life^22,23^. The combined application of AgNO_3_ and PZA has been found to enhance *in vitro* salinity tolerance in tomato plants by disrupting ET action/production and boosting biochemical responses^18^. Due to their effects, these inhibitors are widely used in agriculture and postharvest technology. In order to improve plant tolerance to Cd toxicity, an integrated approach for use of ET inhibitors along with other stress mitigation techniques (like use of metal chelators, organic supplements, and plant growth regulators) may facilitate in better plant growth in Cd-enriched soils.

Mungbean (*Vigna radiata*) is one of the most important legume crops grown in tropical and subtropical areas and, in Pakistan, it is cultivated in kharif season^24^. The crop is a valuable source of iron, vitamins, and zinc. In Pakistan, shortage of clean water is forcing the farmers to use untreated industrial waste water for irrigation. As a results, toxic metals are significantly increased in agricultural fields, leading to entry of noxious pollutants into food chain^1^. Cadmium sensitive mungbean genotypes exhibit reduced root/shoot growth, disturbed plant water contents and stomatal conductance^24^. We hypothesized that an optimum cellular ET concentration is essential for balanced metabolic activities as well as plant development and higher ET concentrations may sometime be harmful for plant growth. In this study, we used ACC (ET precursor) and various inhibitors of ET biosynthesis/perception to draw parallels among ET-induced effects on nutrient ion uptake and biochemical attributes in mungbean genotypes exhibiting differential tolerance to Cd stress.

## Materials and methods

### Experimental layout

**Fig. 1:**
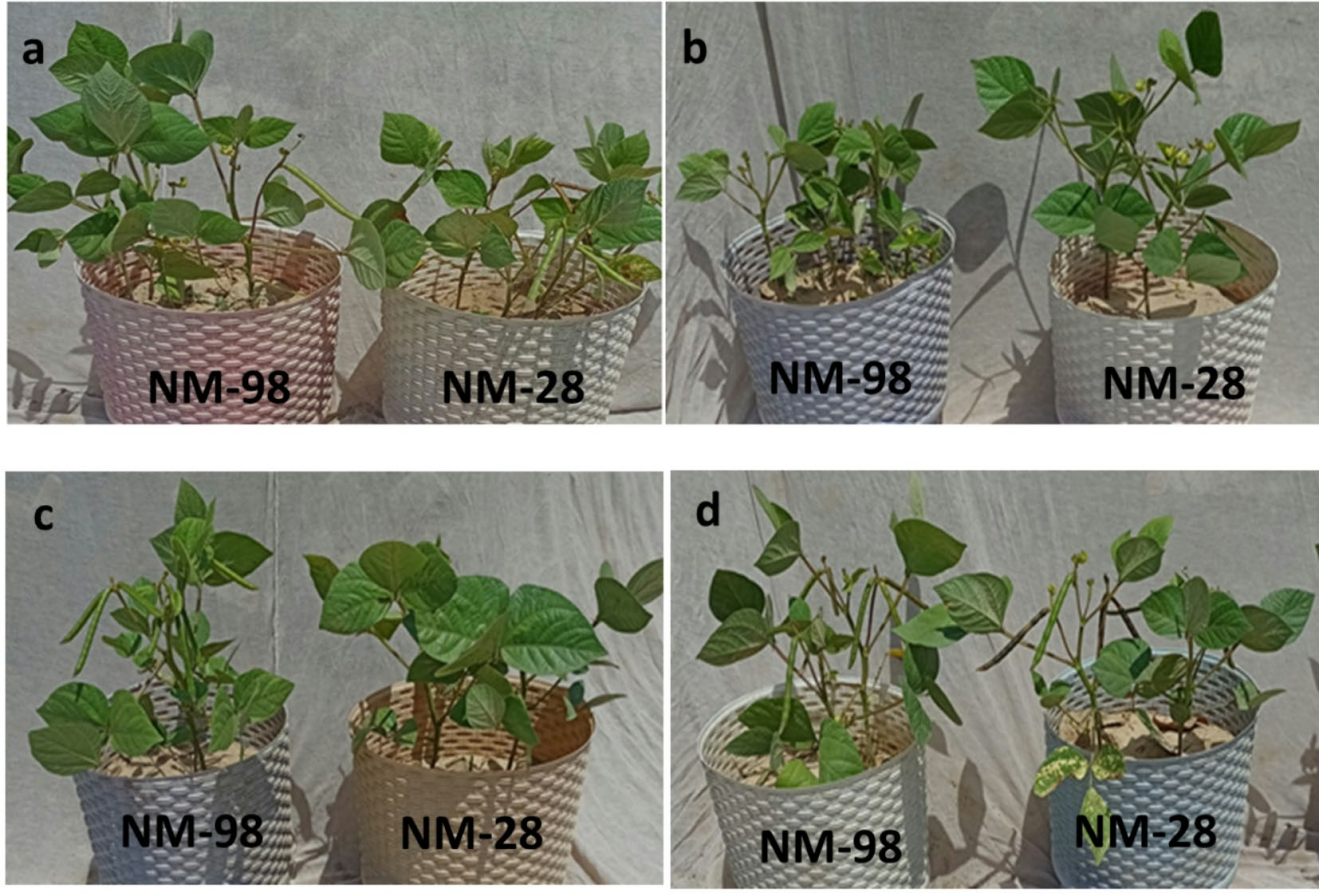
Representative pictures of mungbean plants (NM-98 and NM-28) subjected to various treatments (**a**) 50 µM CdCl_2_ (**b**) 50 µM ACC with 50 µM CdCl_2_ (**c**) 1 mM AVG with 50 µM CdCl_2_ (**d**) 0.25 mM AgNO_3_ with 50 µM CdCl_2_. *ACC* 1-aminocyclopropane-1-carboxylic acid, *AVG* aminoethoxyvinylglycine, *AgNO*_3_ silver nitrate, *CdCl*_*2*_ cadmium chloride.

Seeds of two mungbean genotypes (Cd-tolerant NM-98 and Cd-sensitive NM-28) were obtained from Nuclear Institute of Agriculture and Biology (NIAB), Faisalabad, Pakistan. The stated genotypes were previously characterized in a preliminary lab experiment using various Cd concentrations and the findings regarding Cd-tolerance (data not shown here) were supported by earlier published literature^25,26^. Seeds were thourghly washed, surface sterilized and primed using water (control), ACC (50 µM), AVG (1 mM), PZA (0.5 mM) or AgNO_3_ (0.25 mM). The selected concentrations of ACC, AVG, PZA, and AgNO₃ were based on available literature as well as preliminary experiments conducted in our lab^27,14^. The soil was collected from agricultural field of Faisalabad and found to be slightly alkaline (pH 8.3) with an electrical conductivity (EC) of 4.88 mS cm^− 1^ and an organic matter content (12.8%). It had K (86 mg kg^− 1^), P (16.6 mg kg^− 1^), Fe (2.016 ppm), Co (0.236 ppm) and Mn (0.710 ppm). Seeds were sown in soil-filled pots with five seeds per pot. One week after germination, 50 µM cadmium chloride (CdCl₂) was added to elicit physiological and biochemical responses in mungbean. When plants attained a height of about 5–6 inches above the soil surface, a foliar spray (100 mL) of each i.e. ACC (50 µM), AVG (1 mM), PZA (0.5 mM) and AgNO_3_ (0.25 mM) was applied in two periodic 7-days intervals (Fig. 1a-d). Plants were harvested, after 30 days of first foliar spray, and examined for growth and biochemical attributes.

### Growth assessment

Freshly harvested plants were used to measure root/shoot fresh weight and length using a weighing balance and a measuring scale, respectively. The plants were then oven-dried at 70 °C for 24 h to record root and shoot dry mass.

### Estimation of total chlorophyll

The method of Arnon^28^ was used to determine chlorophyll content. The fresh leaf material was homogenized in an 80% acetone solution and stored at 4 °C overnight. The extract was filtered and centrifuged for 15 min at 3000 g.  Then, a spectrophotometer (Hitachi U-2001, Tokyo, Japan) was used to measure the absorbance of the supernatant at three distinct wavelengths (663, 645, and 480 mm).

### Analysis of total phenolics

A 50% methanol (5 mL) extract of fresh plant material (1 g) was made in order to measure the total phenolic content. A test tube containing 50 µL of plant extract, 1 ml of water, 500 µL of Folin-Ciocalteau reagent, and 2.5 mL of 2% Na_2_CO_3_ were added after 5 min. After incubation for 50 min at room temperature, the absorbance at 710 nm was measured with the help of a spectrophotometer. Gallic acid was utilized to perform a calibration curve, and gallic acid equivalents were used to express the total phenolic concentration^29^.

### Estimation of total soluble proteins

A 0.25 g plant extract was made in 5 mL of phosphate buffer and mixed with 2 mL of Bradford reagent. The mixture was incubated in a water bath for around 30 min. Total soluble protein was calculated by measuring the absorbance of the reaction mixture at 595 nm using a spectrophotometer^30^.

### Determination of hydrogen peroxide

After extracting fresh plant tissue in 5% TCA, 0.1 mL of the plant extract was mixed with 1 mL of phosphate buffer (5 mM) and 2 mL of KI (1 M). According to the method of Velikova et al.^31^, the absorbance of the reaction mixture for H_2_O_2_ was measured at 390 nm.

### Determination of lipid peroxidation

In mungbean leaf tissue, the degree of lipid peroxidation was quantified using the method of Heath and Packer^32^ measuring malondialdehyde (MDA) content using the thiobarbituric acid (TBA) reaction. Plant shoots were homogenized in a POLYTRON ultra-mixer (PT 2000, Switzerland) with 0.1% trichloroacetic acid (TCA). The homogenates were centrifuged at 10,000 g for 5 min, and the supernatant was added to 20% TCA containing 0.5% TBA. The mixture was then heated at 100 °C for 30 min. Quickly stopping the reaction by putting the tubes on ice, the samples were centrifuged once again for 5 min at 10,000 g. Using a spectrophotometer, the supernatant’s absorbance was determined at 532 nm.

### Antioxidant enzymes activity

After homogenizing leaf tissues in 50 mM phosphate buffer (pH 7.8) using POLYTRON ultra-mixture, the samples were centrifuged at 10,000 g for 10 min. Using a spectrophotometer, catalase (CAT) activity in the supernatant was measured by quantifying the decrease in absorbance at 240 nm for 20 s after mixing 100 µL of the sample with 0.75 M H_2_O_2_^33^. In order to measure peroxidase (POD) activity, 0.5 g of frozen plant tissue was mixed with 5 mL of 50 mM KH_2_PO_4_ that was kept at 7 pH. This mixture was centrifuged at 12,000 rpm for 15 min. The supernatant (100 µL) was combined with 3 mL phosphate buffer, 30 µL H_2_O_2_, and 50 µL guaiacol solution. To ascertain the POD activity, the absorbance of the reaction mixture was measured at 436 nm^34^.

### Root nutrients determination

To measure K⁺, Ca²⁺, Mg²⁺, and Cd²⁺ in root samples, element-specific wavelengths [K (766.5 nm), Ca ([142]2.7 nm), Mg (285.2 nm), and Cd (228.8 nm)] are used in the atomic absorption spectrophotometer (AAS). Each element was atomized using an air-acetylene flame in a hollow cathode lamp. Slit width and light current were optimized as per manufacturer’s directions. Briefly, plant roots were oven-dried overnight at 110 °C. Following the addition of conc. H_2_SO_4_, oven-dried samples were incubated for a whole night. After addition of 35% (v/v) H_2_O_2_, the flasks were placed on a heated plate. The samples were left to digest until no further fumes were produced. The mixture was allowed to cool. The process was repeated until the mixture used for digestion became transparent. The transparent blend was diluted, filtered, and stored at 40 °C^35^. The amount of K^+^, Ca^+^, Mg^+^, and Cd^++^ nutrients in the roots was measured by using a flame atomic absorption spectrophotometer (Shimadzu UV/VIS, Kyoto, Japan).

### Statistical analysis

The experiment was conducted by employing a completely randomized design with four replicates for each treatment. The obtained data were subjected to analysis of variance while least significant differences (*p* ≤ 0.05; Fisher’s LSD) was used to record differences among treatment means using a statistical program “CoStat”.

## Results

### Morphological analysis

#### Growth attributes

**Fig. 2:**
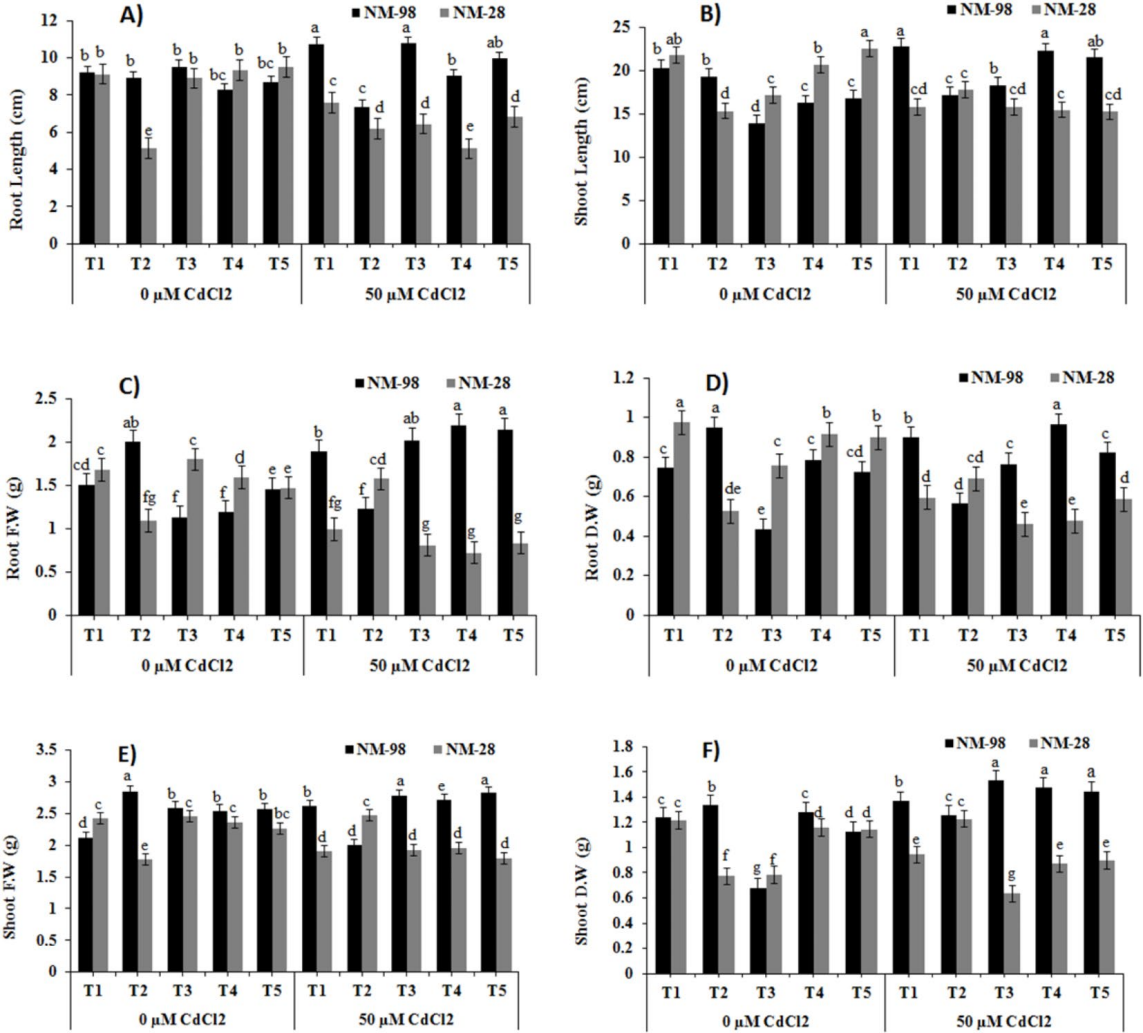
Effects of exogenous application of various treatments on (**A**) root length, (**B**) shoot length, (**C**) root fresh weight, (**D**) root dry weight, (**E**) shoot fresh weight and (**F**) shoot dry weight of mungbean plants (NM-98 and NM-28) subjected to CdCl_2_ stress. control (T1), 50 µM ACC (T2), 1 mM AVG (T3), 0.5 mM PZA (T4) and 0.25 mM AgNO_3_ (T5). Mean values ± SE (*n = 4*) are represented and significant differences (*p* ≤ 0.05) among treatment means are indicated by different lower-case alphabets. *ACC* 1-aminocyclopropane-1-carboxylic acid, *AVG* aminoethoxyvinylglycine, *PZA* pyrazinamide, *AgNO*_3_ silver nitrate, *CdCl*_*2*_ cadmium chloride.

Mungbean cv. NM-28 exhibited a 17% reduction in root length (RL) at 50 µM Cd compared to non-stressed plants. Root length in cv. NM-28, subjected to ACC, was significantly increased (20%) under Cd stress than ACC treated plants without Cd (Fig. 2A). However, this increase was 19% more significant in ACC treated NM-98 genotype than in NM-28 under Cd stress. Under 50 µM Cd stress, exogenously applied AVG, PZA and AgNO_3_ showed a prominent decline of 27%, 45% and 28% in RL of NM-28 while an increase of 13%, 9% and 14% was recorded in cv. NM-98 compared to respective inhibitor-treated plants without Cd stress. The addition of Cd to the soil caused a significant decline (28%) in shoot length (SL) of NM-28 as compared to non-stressed/non-treated plants (Fig. 2B). An increase of 16% was observed in SL of ACC treated plants of NM-28 under Cd stress than respective control. In NM-98, foliar application of ET inhibitors (AVG, PZA, and AgNO₃) under Cd stress led to a substantial increase in SL (16%, 44%, and 41%, respectively) compared to NM-28 plants. A significant drop in root fresh weight (RFW) of cv. NM-98 was noted (38%) after ACC application along Cd stress in comparison to plants grown with ACC but without Cd stress. The plants of NM-28 when subjected to ACC under 50 µM Cd stress revealed 28% higher RFW compared to NM-98. In contrast to NM-98, a minimum decrease (5%) occurred in accordance to Cd stress in RFW of NM-28 after foliar treatments of AVG, PZA as compared to inhibitor treated plants without Cd stress (Fig. 2C). Cadmium stress significantly reduced (39%) root dry weight (RDW) in NM-28 compared to non-stressed plants (Fig. 2D). A maximum reduction of 41% in RDW of NM-98 and elevation of 31% RDW in NM-28 under Cd stress was recorded after ACC application, with respect to ACC treated plants grown in absence of Cd stress. When plant received AVG, a major surge of up to 76% occurred in RDW of NM-98 under Cd stress than plants grown with the same treatment but without Cd stress. Plants grown with Cd-enriched soil showed a maximum reduction (22%) in shoot fresh weight (SFW) in NM-28 than non-stressed/non-treated plants (Fig. 2E). The NM-28 exhibited an increase of 24% and 39% in SFW when plants were exposed to foliar spray of ACC under Cd stress, compared to NM-98 with ACC treated plants grown with and without Cd stress, respectively. An increase of 7%, 7% and 10% in SFW of NM-98 cultivar after application of ET inhibitors (AVG, PZA and AgNO_3_) under Cd stress was recorded when compared with inhibitors treated non-stressed plants. Cadmium addition in soil resulted in a remarkable decrease (22%) in shoot dry weight (SDW) of NM-28 but enhanced SDW (10%) in NM-98 than the non-stressed plants (Fig. 2F). Application of ACC (50 µM) under Cd stress caused a decline of 6% in SDW of NM-98 and a significant increase of 67% in NM-28 than plants grown with ACC and without Cd stress. However, the same genotype (NM-28) exhibited a significant decrease (59%, 41% and 38%) in SDW with foliar treatments of inhibitors (AVG, PZA and AgNO_3_, accordingly) in presence of Cd stress when compared to cv. NM-98 (Fig. 2F).

### Plant biochemical attributes

#### Total chlorophyll content

**Fig. 3:**
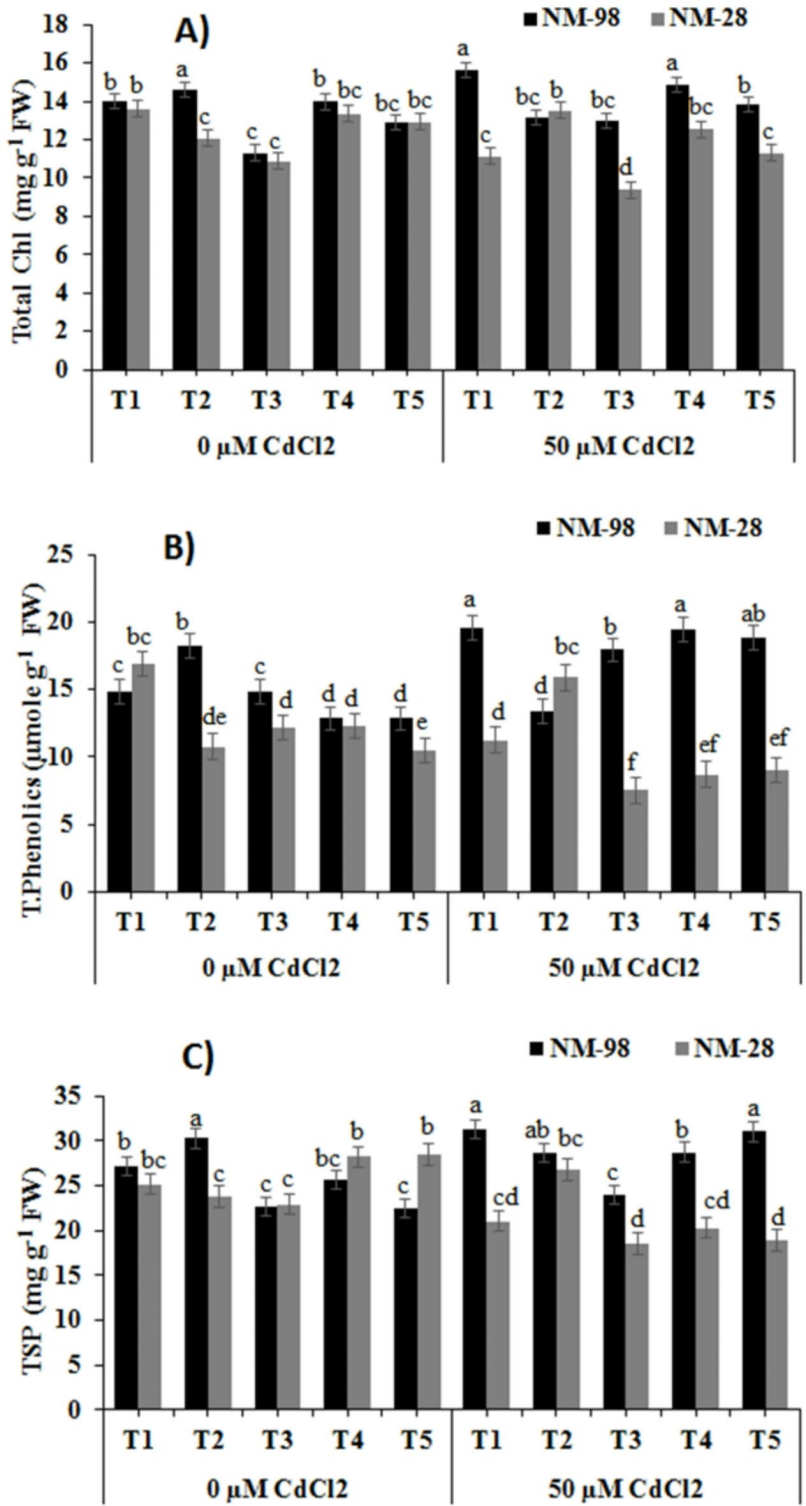
Effects of exogenous application of various treatments on (**A**) total chl (**B**) total phenolics and (**c**) TSP of mungbean plants (NM-98 and NM-28) subjected to CdCl_2_ stress. control (T1), 50 µM ACC (T2), 1 mM AVG (T3), 0.5 mM PZA (T4) and 0.25 mM AgNO_3_ (T5). Mean values ± SE (*n = 4*) are represented and significant differences (*p* ≤ 0.05) among treatment means are indicated by different lower-case alphabets. *ACC* 1-aminocyclopropane-1-carboxylic acid, *AVG* aminoethoxyvinylglycine, *PZA* pyrazinamide, *AgNO*_3_ silver nitrate, *CdCl*_*2*_ cadmium chloride.

In NM-28, ACC treatment under Cd stress led to a significant rise of 12% in total chlorophyll content, whereas a decline up to 10% was observed in NM-98 when compared to the respective control plants (Fig. 3A). The application of ET inhibitors (AVG, PZA, AgNO3﻿) resulted in 38%, 19% and 23% increase, accordingly, in total chlorophyll content in NM-98 compared to NM-28 under Cd stress.

#### Total phenolics and total soluble protein

The plants of NM-98 and NM-28 showed substantial differences in terms of phenolic content under Cd stress. A significant decline of 34% in total phenolics under Cd stress without ACC application was recorded in NM-28 than non-stressed plants (Fig. 3B). In contrast, a major increase of 48% in phenolic content was observed in Cd-srtressed, ACC (50 µM) treated NM-28 plants when compared with non-stressed, ACC treated plants. While, under Cd stress, AVG, PZA and AgNO_3_ treatments showed an increase up to 21%, 51%, and 47%, respectively in total phenolics in NM-98, compared to plants treated with the same inhibitors application in absence of Cd stress. However, in presence of Cd stress, plants of cv. NM-28 when treated with AVG, PZA and AgNO_3_ exhibited a significant decline (58%, 55% and 52%, respectively) in total phenolics compared to cv. NM-98 (Fig. 3B).

Cadmium addition to the soil resulted in a significant reduction (16%) in total soluble protein (TSP) content in NM-28, whereas a significant elevation (15%) was noted in NM-98 compared to respective control plants (Fig. 3C). In comparison to plants grown without Cd, exogenously applied ACC (50 µM) caused a considerable increase (13%) in TSP in NM-28 than ACC-treated plants of same genotype grown without Cd stress. Under Cd stress, a significant decrease of 19%, 28%, 34% was recorded in TSP of NM-28 with AVG, PZA, AgNO_3_ respectively, compared to non-stressed plants treated with the same inhibitors. However, a significant rise of 29%, 41% and 64% was recorded for TSP in NM-98 with the addition of AVG, PZA, AgNO_3_ respectively, compared to NM-28 plants grown with Cd stress (Fig. 3C).

#### Hydrogen peroxide and malondialdehyde

**Fig. 4:**
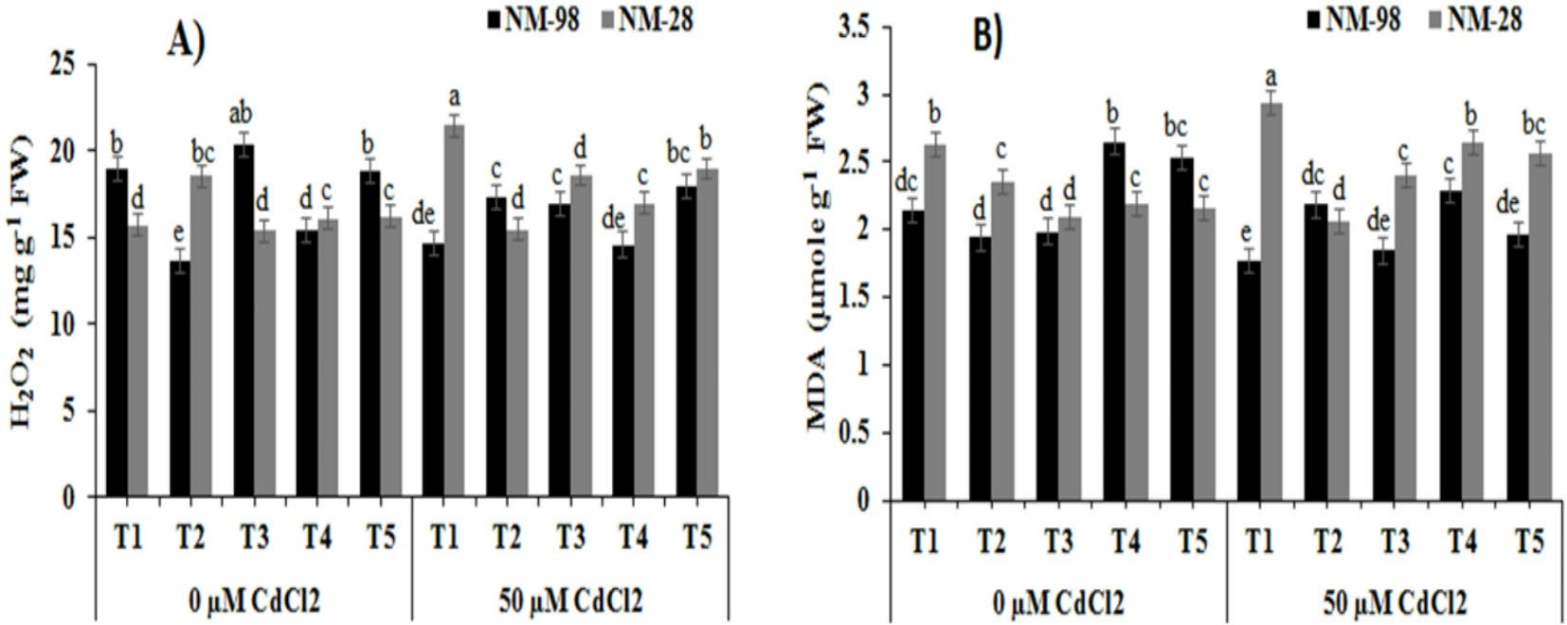
Effects of exogenous application of various treatments on (**A**) hydrogen peroxide (H_2_O_2_) and (**B**) malondialdehyde (MDA) of mungbean plants (NM-98 and NM-28) subjected to CdCl_2_ stress. control (T1), 50 µM ACC (T2), 1 mM AVG (T3), 0.5 mM PZA (T4) and 0.25 mM AgNO_3_ (T5). Mean values ± SE (*n = 4*) are represented and significant differences (*p* ≤ 0.05) among treatment means are indicated by different lower-case alphabets. *ACC* 1-aminocyclopropane-1-carboxylic acid, *AVG* aminoethoxyvinylglycine, *PZA* pyrazinamide, *AgNO*_3_ silver nitrate, *CdCl*_*2*_ cadmium chloride.

As a potent reactive oxygen species (ROS), hydrogen peroxide (H_2_O_2_) serves as an indication of oxidative stress. The cv. NM-98 and cv. NM-28 exhibited a significant rise (22%) and fall (37%) in H_2_O_2_, respectively under Cd stress compared to respective non-stressed/non-treated plants (Fig. 4A). Under Cd stress, levels of H_2_O_2_ remarkably raised (26%) in ACC treated plants of cv. NM-98, while a decrease in H_2_O_2_ content was recorded in Cd-stressed ACC treated NM-28 (16%) compared to ACC treated plants grown without Cd stress. AVG, PZA and AgNO_3_ demonstrated significant upsurge (21%, 5% and 17%, respectively) in H_2_O_2_ concentration in NM-28 under Cd stress with respect to inhibitors treated plants grown without Cd stress.

Under Cd stress, malondialdehyde (MDA) level rises because MDA is produced when polyunsaturated fatty acids in cell membranes break down. In this study, addition of Cd stress in soil increased MDA content in NM-28 while a decline was recorded in NM-98 compared to respective control (Fig. 4B). A decrease of 40% in MDA content was witnessed in cv. NM-98 compared to cv. NM-28 in the presence of Cd stress. The ACC treatment, under Cd stress, resulted in a remarkable decrease (12%) in NM-28 while induced a 13% increase in NM-98 plants when each was compared to respective ACC-treated plants grown without Cd stress. The appication of AVG, PZA and AgNO_3_ (under Cd stress, resulted in reduced (7% 14% and 22%, respectively) MDA content in NM-98 but an enhancement (15%, 21% and 19%, respectively) was recorded in NM-28 compared to respective non-stressed plants grown with inhibitors application.

#### Antioxidant enzyme activity

**Fig. 5:**
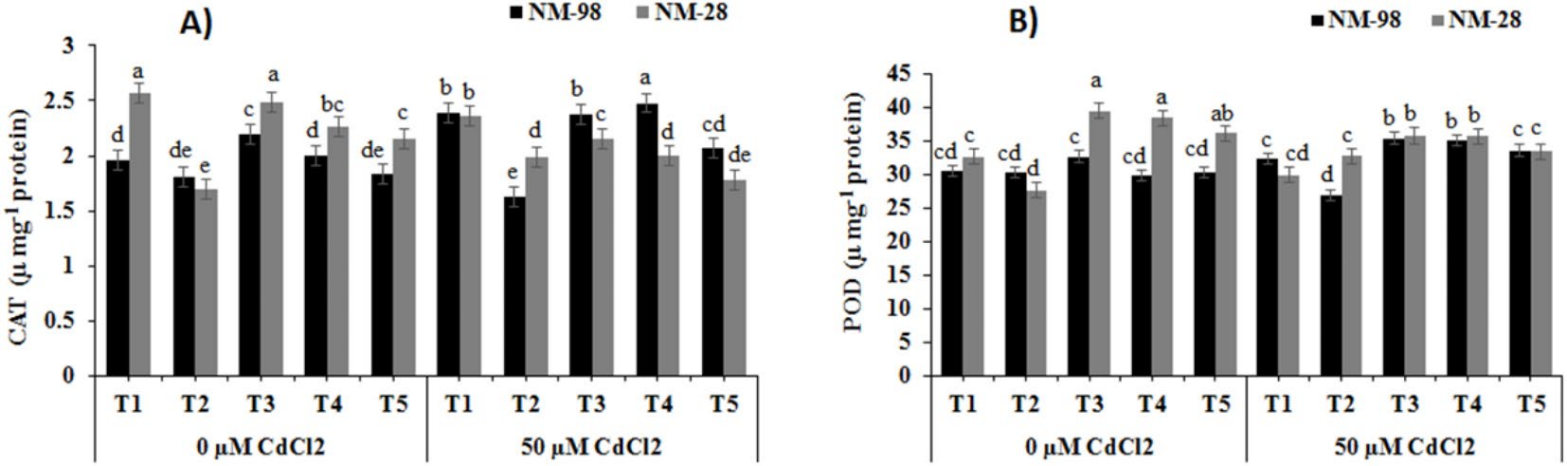
Effects of exogenous application of various treatments on (**A**) peroxidase (POD) and (**B**) catalase (CAT) activity of mungbean plants (NM-98 and NM-28) subjected to CdCl_2_ stress. control (T1), 50 µM ACC (T2), 1 mM AVG (T3), 0.5 mM PZA (T4) and 0.25 mM AgNO_3_ (T5). Mean values ± SE (*n = 4*) are represented and significant differences (*p* ≤ 0.05) among treatment means are indicated by different lower-case alphabets. *ACC* 1-aminocyclopropane-1-carboxylic acid, *AVG* aminoethoxyvinylglycine, *PZA* pyrazinamide, *AgNO*_3_ silver nitrate, *CdCl*_*2*_ cadmium chloride.

Under stress situations, plants have an enzyme-assisted scavenging system for excess produced ROS. Peroxidase (POD) and catalase (CAT) are the two key enzymes in this system. When plants faced Cd stress in soil, 8% decline in CAT activity was recorded in NM-28 compared to non-stressed plants (Fig. 5A). The results depicted a significant rise (22%) in CAT activity in ACC treated NM-28 plants, under Cd stress, compared to NM-98. Furthermore, application of ET inhibitors (AVG, PZA and AgNO_3_), under Cd stress, resulted in increased (8%, 26%, 13%, respectively) CAT activity in NM-98 compared to inhibitor-treated plants grown under non-stress conditions. But similar treatment reduced (13%, 12%, 17%, respectively) CAT activity in NM-28 when each was compared to respective non-stressed inhibitors treated plants.

Cadmium stress negatively influenced POD activity more in NM-28 compared to NM-98 plants. The NM-98 plants indicated 8% higher POD activity compared to cv. NM-28 when subjected to 50 µM Cd stress than control (Fig. 5B). The NM-28 plants depicted maximum POD activity with ACC application under Cd stress which was 18% higher compared to plants grown with ACC application but without Cd stress. All ET inhibitors (AVG, PZA, AgNO_3_) significantly induced POD activity up to 8.4%, 18%, 18% in NM-98 but decreased it up to 9.4%, 7.1%, 8%, respectively in NM-28 when compared to their respective inhibitor treated plants grown without Cd stress (Fig. 5B).

### Plant elemental ions uptake patterns

Nutrient ions uptake patterns serves as an essential metric for evaluating the physiological status of plants. The concentration of several nutritional ions in roots, such as K^+^, Ca^2+^, and Mg^2+^ was measured, and the recorded data is shown in Table 1. Mungbean genotype NM-98 exhibited almost similar Cd uptake in presence and absence of Cd stress. However, NM-28 plants exhibited 26% higher root Cd uptake under Cd stress compared to control plants. With exogenous ACC, Cd-stressed NM-28 roots exhibited 41% less Cd uptake compared to NM-98 roots. In addition, application of ET inhibitors (AVG, PZA and AgNO_3_), under Cd stress, resulted in a significant drop (19%, 20% and 24%, respectively) in root Cd uptake by cv. NM-98 than the inhibitors treated plants without Cd stress. While an upsurge (17%, 15% and 15%) was recorded in root Cd uptake in cv. NM-28 under Cd stress (with ET inhibitors AVG, PZA and AgNO_3_, respectively) when compared to non-stressed control plants (Table 1).

**Table 1:** Effect of ACC (50 µM), AVG (1 mM), PZA (0.5 mM) and AgNO_3_ (1 mM) on root ionic contents of NM-98 and NM-28 mungbean cultivars (cv.) grown with CdCl_2_ (0 and 50 µM) stress. Different lower-case alphabets in the same column indicate ﻿significant differences (p ≤ 0.05) among treatment means.

CdCl_2_	Treatment	cv. NM-98				cv. NM-28			
		Root Cd (µg g^− 1^ DW)	Root K (mg g^− 1^ DW)	Root Ca (mg g^− 1^ DW)	Root Mg (mg g^− 1^ DW)	Root Cd (µg g^− 1^ DW)	Root K (mg g^− 1^ DW)	Root Ca (mg g^− 1^ DW)	Root Mg (mg g^− 1^ DW)
0 µM	Control	7.14 (0.64) ^a^	13.71 (0.83) ^a^	15.00 (0.73) ^b^	5.21 (0.08) ^d^	6.14 (0.55) ^b^	10.71 (0.58) ^a^	12.00 (0.98) ^a^	6.21 (0.09) ^b^
	ACC	6.31 (0.53) ^b^	11.21 (0.59) ^b^	13.22 (0.63) ^c^	5.35 (0.06) ^d^	5.34 (0.43) ^c^	8.32 (0.52) ^b^	9.55 (0.65) ^b^	7.38 (0.14) ^a^
	AVG	5.[142] (0.38) ^c^	10.23 (0.49) ^c^	11.57 (0.43) ^d^	7.11 (0.12) ^c^	6.08 (0.52) ^b^	7.54 (0.38) ^b^	6.53 (0.59) ^c^	5.29 (0.21) ^b^
	PZA	6.75 (0.59) ^b^	12.75 (0.77) ^b^	10.35 (0.33) ^d^	8.34 (0.23) ^b^	5.26 (0. 45) ^c^	6.47 (0.21) ^c^	8.43 (0.57) ^c^	6.32 (0.12) ^b^
	AgNO_3_	5.99 (0.45) ^c^	10.54 (0.55) ^c^	9.27 (0.28) ^e^	10.40 (0.10) ^a^	6.12 (0.45) ^b^	9.36 (0.51) ^a^	11.[142] (0.86) ^a^	8.46 (0.32) ^a^
50 µM	Control	6.97 (0.41) ^a^	15.56 (0.99) ^a^	17.21 (0.79) ^a^	6.75 (0.15) ^c^	7.75 (0.64) ^a^	8.45 (0.44) ^b^	10.34 (0.52) ^b^	5.32 (0.08) ^b^
	ACC	7.23 (0.88) ^a^	9.14 (0.43) ^c^	10.12 (0.35) ^d^	4.11 (0.27) ^d^	4.21 (0.32) ^c^	10.21 (0.54) ^a^	11.22 (0.82) ^a^	8.65 (0.35) ^a^
	AVG	4.37 (0.25) ^d^	12.17 (0.70) ^b^	12.78 (0.54) ^c^	9.20 (0.33) ^b^	7.12 (0.58) ^a^	6.21 (0.25) ^c^	4.28 (0.36) ^d^	4.68 (0.04) ^c^
	PZA	5.39 (0.35) ^c^	13.88 (0.89) ^a^	12.45 (0.51) ^c^	9.77 (0.29) ^b^	6.04 (0.48) ^b^	5.22 (0.23) ^c^	7.11 (0.51) ^c^	5.21 (0.18) ^b^
	AgNO_3_	4.54 (0.29) ^d^	11.75 (0.65) ^b^	11.25 (0.39) ^d^	11.23 (0.17) ^a^	7.20 (0.61) ^a^	7.34 (0.33) ^b^	9.66 (0.63) ^b^	6.23 (0.08) ^b^

*ACC* 1-aminocyclopropane-1-carboxylic acid, *AVG* aminoethoxyvinylglycine, *PZA* pyrazinamide, *AgNO*_3_ silver nitrate, *CdCl*_2_ cadmium chloride.

Addition of Cd in the soil caused a prominent reduction (46%) in root K uptake in cv. NM-28 than cv. NM-98. Contrarily, a significant positive influence of ACC application was noted in terms of improved K contents (12% higher) in NM-28 genotype under Cd stress than cv. NM-98. With Cd stress, a reduction of 18%, 19% and 22% was recorded in K uptake in NM-28 in accordance to ET inhibitors i.e. AVG, PZA and AgNO_3_, respectively than the non-stressed plants with ET inhibitors (Table 1).

Root Ca uptake indicated a significant increase of 15% in NM-98 but a considerable decline (14%) was noted in NM-28 plants grown under Cd stress without any foliar/inhibitor treatment than the non-stressed control plants. The two genotypes responded differently in terms of root Ca content following the application of ACC along with Cd. Exogenous application of ACC (50 µM) with Cd stress minimized root Ca uptake up to 23% in NM-98 but raised it significantly (17%) in NM-28 compared to their respective controls (without Cd but with ACC). Inhibitors application also produced antagonistic effects in both genotypes. Root Ca uptake was enhanced considerably (10%, 20% and 21%) by ET inhibitors in NM-98 but suffered a significant drop (34%, 16% and 15%) in NM-28 with Cd toxicity when compared to respective inhibitors (AVG, PZA AgNO_3_, respectively) treated control plants (without Cd stress).

Under Cd stress, a significant (30%) increase in magnesium (Mg) uptake was recorded in NM-98, while NM-28 plants exhibited a significant reduction (14%) than respective control plants (grown without Cd stress) (Table 1). Under Cd stress, with exogenous ACC application, a remarkable rise (17%) was witnessed in Mg uptake for NM-28, with respect to non-stressed ACC treated plants. Application of ET inhibitors (AVG, PZA and AgNO_3_) with Cd stress showed quite opposite results to ACC treatment i.e. elevated Mg uptake (29%, 17% and 8%, respectively) in cv. NM-98, but dropped Mg uptake to a significant extent (12%, 18% and 26%, respectively) in NM-28 plants than the non-stressed plants subjected to inhibitors (Table 1).

## Discussion

One of the major environmental barriers to agricultural productivity and food security is cadmium (Cd) stress. A great extent of yield loss (20–50%) of agricultural crops is caused by this expanding menace^36^. Fresh and dry biomass declined significantly under Cd stress in sensitive cultivars (NM-28) than tolerant cultivar (NM-98)^25,26^, as evidenced by the wilting of leaves, which was probably caused by a decrease in root water absorption^37^. In the present study, exogenous 50 µM ACC (1-aminocyclopropane-1-carboxylic acid) administration improved plant fresh and dry weight in NM-28 (a Cd sensitive genotype) despite the presence of Cd in soil depicting that ACC assisted NM-28 plants in ET synthesis, thereby increasing stress tolerance. However, the same treatment negatively affected NM-98, likely disrupting endogenous ET levels and weakening its stress tolerance. These findings were similar as recorded for *Nelumbo nucifera*^38^ plants exposed to exogenous ACC application under Cd stress. It appeared that ACC application along Cd stress increased oxidative stress, H_2_O_2_ production, disruption of the chloroplast structural system, and possibly chlorophyll enzyme activity, leading to a notable decrease in growth parameters in Cd tolerant mungbean^39^. Furthermore, present study findings revealed that exogenous application of ET inhibitors (AVG, PZA, and AgNO_3_) increased plant growth in NM-98 and lowered in NM-28. The application of ET inhibitors (AVG and PZA) inhibited stress-induced ET biosynthesis in NM-98 by interfering with the enzyme activities of ACC synthase (ACS) and ACC oxidase (ACO), subsequently enhancing plant biomass. On the other hand, AgNO_3_ reduces ET action/perception and maximizes the polyamines production. It was noticeable that polyamines promote plant growth and cell proliferation in tomato plants under abiotic stress^18^. Therefore, AgNO_3_ might have improved abiotic tolerance by stimulating polyamines biosynthesis.

The most basic physiological activity in plants is photosynthesis, which produces the energy needed for growth and metabolism^40^. Under Cd-stressed conditions, chlorophyll content and photosynthetic apparatus reduced along with plant biomass^36^. However, few investigations looked into whether ACC bypasses ET perception or plays a non-canonical role independent of ET signaling. In this study, analysis of plant biochemical attributes indicated that ACC treated NM-28 resulted in improved total chlorophyll content under Cd stress compared to NM-98. The current study demonstrated that ET inhibition acted as a negative regulator of chlorophyll synthesis in Cd-sensitive genotypes as recorded for NM-28 and reported earlier^27^. But, inhibitors (AVG, PZA and AgNO_3_) acted antagonistically in NM-98 by suppressing the catalytic activity of ACO (ACC oxidase), ACS (ACC synthase) and ET perception, respectively, which in turns declined ET synthesis and improved total chlorophyll. The balanced chlorophyll content observed in Cd tolerant NM-98, under Cd stress following AVG, PZA and AgNO_3_ exposure is most likely due to improved nutrient availability^39^, which contributed to the mitigation of Cd-induced ET-mediated stress responses. Previous studies have confirmed that accumulation of ET-inhibitors in leaves disrupted the thylakoid membrane structure and decreased chlorophyll levels, ultimately hindering plant growth in Arabidopsis and tomato^41,42^, which is consistent with the observations recorded for cv. NM-28 in the current study. Additionally, the findings recorded for cv. NM-98 in the current study aligns with those reported by Djanaguiraman et al.^43^ where the authors reported reduced ET production as well as perception, lowered ROS accumulation, increased antioxidant enzyme activity and preserved plant photosynthetic pigment concentrations upon application of 1-MCP (1-methylcyclopropene) and AVG.

Phenolic chemicals are recognized as antioxidants as well as specialized metabolites. Their accumulation often requires elicitors, which function as molecular cues in plant stress responses. Low phenolic content concentrations in NM-28 (compared to NM-98) indicated that an excess of Cd might have hampered the anti-oxidative system’s reactions, making it impossible for plants to synthesize phenolics and other related chemicals as previously reported in *Zea mays*^44^. However, a reduction in phenolic components upon ACC application was recorded in NM-98 but not in NM-28. Similar findings were obtained in black carrot roots when plants were exposed to ethepone, a source of ET^45^. Exogenously applied ET inhibitors acted antagonistically (improved total phenolics in NM-98 and declined in NM-28) to ACC application. These inhibitors possibly reduced or inhibited ET synthesis/perception and assisted NM-98 oxidative machinery to counteract the harmful consequences of Cd toxicity as well as elevated cellular ET levels. Exogenous application of ET inhibitors under abiotic stress conditions lead to increased accumulation of phenolic compounds in mungbean plants. This enhancement in phenolic content might contribute to improved antioxidant capacity, aiding in mitigation of oxidative damage and promoting stress tolerance. However, the extent of these effects may vary depending on the plant species, type of abiotic stress, and use of a specific ET-inhibitor.

A balance concentration of cellular proteins is required for optimum plant growth. Cadmium stress can disrupt a balance between total soluble proteins (TSP) of general-function and stress-responsive proteins^45^. Cadmium addition resulted in reduced TSP and results are aligned with previous reports^46^. The results of the current study revealed that exogenously applied ACC, under Cd stress, might have initiated signal cascades by an unidentified mechanism, that raised the amount of soluble proteins in NM-28 and enhanced their antioxidant enzyme activity. Similarly, application of inhibitors AVG, PZA and AgNO_3_ showed maximum upsurge in TSP levels in NM-98 compared to NM-28. The obtained outcomes might be due to enhanced nutrient absorption capacity and antioxidant activity that, probably, suppressed ACO enzyme activity (responsible of ET synthesis) and ET perception, which were consistent with those of earlier studies^15^. Inhibition of ET-biosynthesis promoted accumulation of heat shock proteins (HSPs) and antioxidant enzymes including SOD (superoxide dismutase) and CAT, which helped to mitigate oxidative stress and maintain protein homeostasis in soybean^43^. A decrease in protein content was linked to the acceleration of leaf senescence caused by ET, and ET inhibitors (AVG, AgNO_3_) assisted to delay senescence and maintain TSP levels in leaves and other tissues of *Solanum chilense* by inhibiting ET activity under abiotic stress^47^.

Cadmium stress generally led to an increase in reactive oxygen species (ROS) in plant leaves causing oxidative damage to membrane components and organelles in cells^46^. The detection of lipid peroxidation by MDA content is a frequently used biomarker of membrane damage because ROS interacted with phospholipids and fatty acids, causing lipids to be oxidized and raising the quantity of MDA^48^. In the present study, application of ACC under Cd stress depicted more decrease in H_2_O_2_ and MDA contents in NM-28 compared to NM-98. An optimum cellular ET concentration decreased oxidative stress by decreasing H_2_O_2_ and TBARS levels and boosting photosynthetic activity^49^. It was found that mungbean NM-28 treated with ACC, under Cd stress, induced ET production that reduced Cd induced oxidative damage by decreasing H_2_O_2_ and MDA level and electrolyte leakage thereby enhancing the antioxidants enzyme activity. However, when genotypes were exposed to ET-inhibitors (AVG, AgNO_3_ and PZA), the negative effects of Cd were reduced, as revealed by less lipid peroxidation (low MDA content) and low H_2_O_2_. This mitigation of Cd-induced damage was better in NM-98 than NM-28. This aligned with prior research in *Brassica napus*, where AgNO₃ and other ET inhibitors improved abiotic stress tolerance by reducing oxidative damage^50^. By lowering MDA and H_2_O_2_ levels in plants subjected to abiotic stress, ET inhibitors (AgNO_3_ and AVG) were proved to be good in preventing oxidative damage in *Brassica napus*^50^ and soybean^43^. Their capacity to inhibit ET perception or synthesis promoted antioxidant enzyme activity, controls ROS metabolism, and preserves membrane integrity^24^.

Antioxidants play a more important part in respiration at the mitochondrial site and in the process of photosynthesis at chloroplasts. Elevated production of ROS or cyanide accumulation﻿, under stressful conditions, can disrupt activity of antioxidant enzymes such as SOD, CAT and ascorbate peroxidase (APX)^51^. Among these, CAT and POD are the most important enzymatic antioxidants found in plants^51^. Under Cd stress, ACC application resulted in enhanced CAT and POD activity in NM-28, while a reduction was recorded in NM-98. It seemed that foliar ACC, along with Cd, increased the amount of ET from its optimum cellular level, which impaired plant’s ability to combat the oxidative stress in NM-98. Previous studies had shown that ET (produced from ethephon) increased antioxidant enzyme activity at an ideal level and detoxified excess ROS; however, excess levels of ET itself caused reductions in important oxidative enzyme activities like CAT, glutathione/ascorbate reductases, POD, and SOD^49^. In the current study, the use of ET inhibitors (AVG, PZA and AgNO_3_) increased POD and CAT in NM-98 as compared to NM-28 in response to Cd stress. This suggested that NM-98 could better regulate Cd-induced ROS accumulation due to the action of ET-inhibitors, which helped plants to maintain ET levels and thus reduced oxidative stress. This outcome was in line with earlier findings^11^ where an increase in the activity of antioxidant enzymes was recorded in rice exposed to salt stress and ET-inhibitors. A study by Zarei and Ehsanpour^18^ demonstrated that AgNO_3_ and PZA treated tomato plants maintained higher levels of photosynthetic pigments, had enhanced antioxidant enzyme activity, and exhibited less malondialdehyde (MDA) content, indicating reduced lipid peroxidation under abiotic stress.

Increased plant growth is indicative of improved assimilation rate and effective nutrient uptake. Enhanced dry matter and minerals are generally the outcome of improved nutrient absorption^36^. Cadmium is a divalent cation and it competed with other nutrients for transport at the absorption site which reduces intake of essential elements from the soil such as Zn, Mn, Ca, Mg, and Fe^18^. The antagonistic relationship between the concentration of Cd in soil solution and the accumulation of vital nutrients varied depending on the genotype of the plant. Contrasting effects of foliar application of ACC on nutrient uptake by two mungbean genotypes were recorded. In Cd tolerant cultivar (NM-98), ACC reduced the uptake of essential nutrients from soil, while an increased nutrient uptake was recorded in Cd sensitive NM-28. These differences could be attributed to the role of ET in stress responses. It is likely that NM-98 was producing optimum ET and exogenous ACC application further stimulated ET production thereby converting ET into a stress inducer rather than a protector. Conversely, NM-28 was not sufficiently producing ET, and exogenous ACC helped NM-28 achieve balanced ET production, thus improving nutrient uptake and better growth under Cd stress^25,26^. Our research revealed a positive correlation between total biomass and the uptake of Ca^2+^, Mg^2+^, and K^+^ in mungbean NM-98 compared to NM-28 after exposure to various ET inhibitors under Cd stress. This suggested that ET inhibitors (AVG, PZA and AgNO_3_) assisted NM-98 in acclimating to soil conditions by improving nutrient acquisition. Specifically, ET inhibitors enhanced essential nutrient acquisition by modifying root physiology^52^, altering root exudation, and enhancing nutrient cycling by suppressing ET biosynthesis and perception in Cd tolerant genotype (NM-98). The results were in agreement with earlier literature^18^ where it was demonstrated that ET inhibitors i.e. PZA and AgNO_3_ treated tomato had higher assimilation rate leading to higher nutrient uptake and dry matter.

## Conclusion

Negative effects of exogenous ACC application recorded in NM-98 indicated that Cd-tolerance of this genotype was associated with an optimum ethylene (ET) concentration. Possibly, availability of excess ACC resulted in enhanced cellular ET concentrations and initiated abiotic stress-induced growth retardations in NM-98. In contrast, Cd-sensitive genotype “NM-28” was low in ET levels and thus exogenous ACC helped to sustain optimal ET levels resulting in an improved growth, nutrient uptake, and oxidative stress tolerance. The reversal of the ACC-mediated growth and biochemical patterns, in both genotypes, by ET inhibitors (AVG, PZA, AgNO₃) further confirmed the significance of cellular ET levels under normal and Cd-stressed conditions. Among the various ET inhibitors, AgNO₃ induced effects were more profound in both the genotypes indicating that ET perception was equally important factor for balanced cellular metabolism, plant growth and development.

## Electronic supplementary material

Below is the link to the electronic supplementary material.

Supplementary Material 1

## Data Availability

All data generated or analyzed during the study are included in this article.
